# A randomized, double-blind, placebo-controlled study of flexible doses of levomilnacipran ER (40–120 mg/day) in patients with major depressive disorder

**DOI:** 10.3109/21556660.2014.884505

**Published:** 2014-01-16

**Authors:** Carl P. Gommoll, William M. Greenberg, Changzheng Chen

**Affiliations:** Forest Research Institute, Jersey City, NJUSA

**Keywords:** Levomilnacipran ER, Major depressive disorder (MDD), Serotonin and norepinephrine reuptake inhibitor (SNRI), Antidepressant clinical trial

## Abstract

**Objective:**

Levomilnacipran ER is a potent and selective serotonin and norepinephrine reuptake inhibitor (SNRI) approved for the treatment of major depressive disorder (MDD). Efficacy and safety have been evaluated in five Phase II/III studies, four of which met the pre-specified primary efficacy outcome. Results of the negative trial (ClinicalTrials.gov NCT00969150) are reported here.

**Methods:**

A Phase III randomized, double-blind, placebo-controlled trial comparing flexible-dose levomilnacipran ER 40–120 mg/day with placebo was conducted in outpatients with MDD. Patients met the DSM-IV-TR criteria for MDD, had a current episode of depression of at least 4 weeks’ duration, and a Montgomery-Åsberg Depression Rating Scale (MADRS) total score ≥30. The study comprised a 1-week, single-blind, placebo lead-in, 8-week double-blind treatment, and a 2-week down-taper. The primary and secondary efficacy measures were change from baseline to Week 8 in MADRS and Sheehan Disability Scale (SDS) total scores, respectively, analyzed using a mixed-effects model for repeated measures approach. Safety outcomes included adverse events (AEs), laboratory and vital sign measures, the Columbia-Suicide Severity Rating Scale, and the Arizona Sexual Experiences Scale (ASEX).

**Results:**

Three hundred and fifty-five patients received the study drug and had ≥1 post-baseline MADRS total score assessment (ITT Population); 81.9% of placebo and 77.1% of levomilnacipran ER patients completed the study. For levomilnacipran ER vs placebo, MADRS (−15.7 vs −14.2) and SDS (−8.8 vs −8.2) total score improvements, and rates of MADRS response (38.5% vs 34.8%) and remission (25.3% vs 23.8%) were numerically greater but differences were not statistically significant. Levomilnacipran ER was generally well tolerated. More levomilnacipran ER patients vs placebo reported AEs; the most common AEs for levomilnacipran ER were nausea (17%) and headache (16%). Mean changes in most safety measures were small and similar between groups. There were no meaningful differences in total ASEX scores between groups.

**Limitations:**

Short duration of treatment, inclusion and exclusion criteria, and lack of an active comparator.

**Conclusion:**

Numerical improvements for levomilnacipran ER vs placebo were detected in this study, but the differences were not statistically significant; levomilnacipran ER was generally well tolerated.

## Introduction

Major depressive disorder (MDD) affects ∼120 million people worldwide, including nearly 15 million American adults each year; it is a leading cause of illness-related disability^1,2^. Response rates in acute treatment trials remain relatively low, with as many as 30–50% of patients failing to respond adequately to the first or second medication administered^3–5^. Poor tolerability and adverse events (AEs) contribute to low response rates by increasing the likelihood of medication non-compliance and premature discontinuation, impeding the goal of achieving antidepressant therapy of adequate dose and duration^6^. AEs that are considered more bothersome, such as sexual dysfunction and weight gain, are significantly associated with treatment discontinuation^7,8^. Compounds that selectively inhibit the reuptake of serotonin and/or norepinephrine have been shown to be effective in the treatment of MDD^9–11^, but substantial unmet needs still remain with currently available antidepressants.

Levomilnacipran (1*S*, 2*R*-milnacipran) is a potent and selective serotonin and norepinephrine reuptake inhibitor (SNRI) that is approved for the treatment of MDD in adults; an extended-release (ER) formulation was developed to allow for once-daily dosing. *In vitro* studies have shown that levomilnacipran has ∼2-fold greater potency for norepinephrine relative to serotonin reuptake inhibition^12^ and compared with the SNRIs duloxetine^12^, venlafaxine^12^, or desvenlafaxine it is 10-fold more selective for norepinephrine vs serotonin reuptake inhibition^13^. Levomilnacipran is the more active enantiomer of milnacipran, an SNRI that is approved for the treatment of fibromyalgia in the US (prescribing information: Savella [milnacipran hydrochloride], 2011; Forest Laboratories, Inc: St Louis, MO). Milnacipran is not approved for the treatment of MDD in the US; however, it is approved for this indication in many other countries. Milnacipran studies in MDD were conducted more than a decade ago and no head-to-head trials with levomilnacipran ER have been performed. As such, no valid comparison of levomilnacipran ER and milnacipran can be made based on the clinical data.

The clinical development program for levomilnacipran ER for the treatment of MDD has included four Phase II/III, randomized, double-blind, placebo-controlled, flexible-^14,15^ or fixed-dose^16,17^ trials in which the pre-specified primary efficacy endpoint was met. Levomilnacipran ER at the doses evaluated was generally safe and well tolerated. In an additional flexible-dose study conducted concurrently with the positive trials, levomilnacipran ER failed to achieve statistically significant separation from placebo on the primary efficacy measure; the efficacy, safety, and tolerability results of this study (ClinicalTrials.gov: NCT00969150) are reported here.

## Patients and methods

This Phase III randomized, double-blind, controlled trial comparing flexible doses of levomilnacipran ER 40–120 mg/day with placebo was conducted in adult outpatients with MDD. The study was conducted at 24 centers in the US between September 2009 and October 2010 in full compliance with US Food and Drug Administration (FDA) guidelines for Good Clinical Practice and in accordance with the Declaration of Helsinki. Each center’s institutional review board approved the study, and all patients provided written informed consent.

### Study design

Following a 1-week, single-blind, placebo lead-in, patients who continued to meet eligibility criteria were randomized (1:1) to 8 weeks of double-blind treatment with levomilnacipran ER or placebo. Patients were randomized by a computer-generated list of numbers and assigned to identically appearing levomilnacipran ER or placebo. Investigators and patients were blinded to allocation of the investigational product throughout treatment and down-taper periods. The blind was maintained via a secured randomization code list and was broken only in the case of emergency; unblinding disqualified a patient from further study participation.

All patients randomized to levomilnacipran ER received 20 mg on Days 1 and 2, and 40 mg beginning on Day 3. At the end of Week 1 or Week 2, the dose could be increased to 80 mg/day based on patient response and tolerability. At the end of Week 4, the dose could be increased to 80 mg/day for patients who were previously receiving 40 mg/day or to 120 mg/day for patients who were previously receiving 80 mg/day. No dose increase was permitted after Week 4, but dosage could be decreased to the previous level at any time if the Investigator determined that there were significant tolerability issues. After 8 weeks of treatment, or at premature discontinuation of the study, patients entered a 2-week down-titration period in which doses were gradually down tapered every 3–7 days unless the Investigator felt this was clinically not indicated.

### Key inclusion criteria

Male and female outpatients who were 18–80 years of age, inclusive, and met the *Diagnostic and Statistical Manual of Mental Disorders*, Fourth Edition, Text Revision (DSM-IV-TR)^18^ criteria for MDD were eligible to participate; the diagnosis of MDD was confirmed by the Mini International Neuropsychiatric Interview (MINI)^19^. Patients had a current episode of depression of at least 4 weeks’ duration, and a clinician-rated Montgomery-Åsberg Depression Rating Scale (MADRS)^20^ total score ≥30 at screening and after the 1-week placebo lead-in period. Patients who were included in the trial had normal results on physical examination, clinical laboratory tests, and electrocardiograms (ECGs), or abnormal results that were judged not clinically significant by the Investigator.

### Key exclusion criteria

Patients were excluded from the trial if they had: a DSM-IV-TR primary Axis I diagnosis other than MDD within 6 months of screening (co-morbid generalized anxiety disorder, social anxiety disorder, and/or specific phobias were allowed); a history of a manic/hypomanic episode or other significant psychiatric disorder (e.g., schizophrenia, depressive episode with psychotic features, obsessive-compulsive disorder), cognitive disorder, or substance abuse/dependence within 6 months of the study; any concurrent medical condition that could interfere with the conduct of the study, confound the interpretation of study results, or endanger the patient’s well-being (e.g., clinically significant systolic and/or diastolic blood pressure readings, central nervous system, or cardiovascular disorders); a history of non-response to two or more adequate treatment trials with antidepressants; a history of intolerance or hypersensitivity to other SNRIs, SSRIs, or selective noradrenergic reuptake inhibitors; or previous participation in any investigational study of milnacipran or levomilnacipran.

Current treatment with any drug with psychotropic activity (except for eszopiclone, zolpidem, or zaleplon for sleep) was prohibited. Females of child-bearing potential who were pregnant, breastfeeding, or not currently using a medically acceptable method of contraception were excluded. Patients with a significant risk of suicide, identified as a suicide attempt within the past year, score ≥5 on MADRS Item 10 (Suicidal Thoughts), or significant risk based on Investigator judgment or information from the Columbia-Suicide Severity Rating Scale (C-SSRS)^21^, were also ineligible to participate.

### Efficacy assessments

The primary efficacy measure was the MADRS, which was assessed at screening (Week −1), baseline (Week 0) and Weeks 1, 2, 4, 6, and 8; the secondary efficacy measure was the Sheehan Disability Scale (SDS) (Weeks 0, 4, 6, and 8)^22^. Additional efficacy outcomes included the 17-item Hamilton Rating Scale for Depression (HAMD_17_) (Weeks −1, 0, 1, 2, 4, 6, 8)^23^, and the Clinical Global Impressions-Severity (CGI-S) (Weeks 0, 1, 2, 4, 6, 8) and -Improvement (CGI-I) Scales^24^ (Weeks 1, 2, 4, 6, 8).

### Safety assessments

Spontaneously reported or observed AEs were assessed at all post-screening study visits; AEs were evaluated in terms of intensity (mild, moderate, or severe) and possible relationship to the study drug. Additional safety evaluations included physical examinations (Weeks −1, 8), vital sign monitoring (every visit), clinical laboratory evaluations (Weeks −1, 4, and 8), and ECGs (Weeks −1, 1, 4, and 8).

The 5-item Arizona Sexual Experiences Scale (ASEX)^25^ was used to assess sexual experiences with respect to libido, psychological and physiological arousal, ability to attain orgasm, and satisfaction with orgasm (baseline, Weeks 4 and 8). Each item on the scale is rated from 1–6, with higher scores indicating greater sexual dysfunction. Categorical evaluation of sexual dysfunction or no sexual dysfunction at baseline and endpoint is provided; change from baseline in ASEX total score is also presented.

The C-SSRS, used to assess the severity of suicidal behavior and ideation, was completed at screening Visit 1 to obtain lifetime history of suicidal ideation and behavior. At all other visits, the C-SSRS was completed to evaluate ideation and behavior since the previous visit. Suicidal ideation is classified on a scale from 1 (wish to be dead) to 5 (active suicidal ideation with specific plan and intent); suicidal behavior is classified on a scale from 0 (no suicidal behavior) to 4 (actual attempt).

Health outcomes were assessed using the Physical Component Summary (PCS) and Mental Component Summary (MCS) scores of the Short Form-36 Health Survey (SF-36)^26^; scores are based on general US population norms (mean = 50; SD = 10), with higher scores indicative of better health.

### Statistical analysis

Safety analyses were based on the Safety Population, which consisted of all patients who were randomized and received at least one dose of study drug. Efficacy analyses were based on the Intent-to-Treat (ITT) Population, which consisted of all patients in the Safety Population who also had at least one post-baseline MADRS total score assessment. Demographic and baseline efficacy outcomes were compared between treatment groups using an analysis of variance (ANOVA) model, with treatment group and study center as the factors for continuous variables, and the Cochran-Mantel-Haenszel test, controlling for study center, for categorical variables.

The primary efficacy measure was change from baseline to Week 8 in MADRS total score; the primary analysis used a mixed-effects model for repeated measures (MMRM) approach with treatment group, pooled study center, visit, and treatment-group-by-visit interaction as factors, and baseline value and baseline-by-visit interaction as covariates. A sample size of 180 patients in each of the two treatment groups was estimated to provide 93% power to detect an effect size of 0.38 between the placebo and levomilnacipran ER 40–120 mg/day groups based on an MMRM model.

Sensitivity analyses for the primary efficacy measure were carried out using the last observation carried forward (LOCF) approach and the pattern mixture model (PMM)^27^. For the LOCF approach, the between-treatment group comparison was performed using an ANCOVA model with treatment group and pooled study center as factors and the baseline MADRS total score as a covariate. For the PMM approach, a pattern-mixture model based on non-future dependent missing value restrictions was performed to assess the robustness of the primary MMRM results.

The secondary efficacy measure, change in SDS total score from baseline to Week 8, was analyzed similarly to the primary efficacy measure; sensitivity analysis was performed using the LOCF approach. Additional efficacy measures included change from baseline to Week 8 on HAMD_17_ total score and CGI-S score, and CGI-I score at Week 8; analyses were similar to those used for the primary efficacy measure. By-visit analyses were performed for all efficacy measures using MMRM and LOCF approaches. Response (MADRS ≥50% improvement from baseline and CGI score ≤2) and remission (MADRS total score ≤10) rates at Week 8 were analyzed using a logistic regression model with treatment group and the corresponding baseline score as explanatory variables for the LOCF approach only; the baseline CGI-S score was used for CGI-I responder analysis. Descriptive statistics were provided for safety measures. All statistical tests were 2-sided hypothesis tests performed at the 5% level of significance; all confidence intervals (CIs) were 2-sided 95% CIs.

## Results

A total of 362 patients were randomized to receive treatment; of these, 357 patients received ≥1 dose of study drug (Safety Population) and 355 patients received study drug and had ≥1 post-baseline MADRS total score assessment (ITT Population) (Table 1). Overall, 81.9% of placebo- and 77.1% of levomilnacipran ER-treated patients completed the study; reasons for premature discontinuation from the study are shown in Table 1. The most frequent reasons for discontinuation were withdrawal of consent and protocol violation. Patients in the placebo group were numerically more likely to withdraw consent, so it did not appear that undetected AEs were included in this category or were the true reason for discontinuation. AEs led to the discontinuation of 14 patients in the levomilnacipran ER group and four patients in the placebo group, a difference that was statistically significant (*p* = 0.0147).

**Table 1.** Patient disposition.

	Placebo	Levomilnacipran ER 40–120 mg/day
Safety population, *n*	182	175
Intent-to-treat population, *n*	181	174
Completed study, % (safety population)	81.9	77.1
Reason for premature discontinuation		
Adverse event	2.2	8.0*
Protocol violation	4.9	6.9
Withdrawal of consent	7.1	5.1
Lost to follow-up	2.7	2.3
Insufficient therapeutic response	0.5	0.6
Other	0.5	0

*p < 0.05. ER, extended-release.

There were no statistically significant differences between treatment groups with respect to baseline demographic characteristics or MDD disease history (Table 2). Most patients (72%) had a history of recurrent major depression and the mean duration of MDD was ∼11 years; mean age at onset was 32 years. Even though most patients had recurrent depression, only about half of all patients had received prior antidepressant therapy (57% placebo; 47% levomilnacipran ER). Nearly one third of patients with previous antidepressant use either had a poor response or non-response to prior therapy. Mean baseline scores on the secondary and additional efficacy measures were also similar between treatment groups (Table 3).

**Table 2.** Patient characteristics (safety population).

	Placebo (*n* = 182)	Levomilnacipran ER 40–120 mg/day (*n* = 175)
Demographic characteristics		
Age, mean (SD), years	43.7 (13.3)	42.8 (12.9)
Sex, % women	63.7	56.6
Race, % white	81.9	76.0
BMI, mean (SD), kg/m^2^	28.9 (5.7)	28.7 (5.4)
Major depressive disorder (MDD) disease characteristics
Age at onset, mean (SD), years	32.2 (14.2)	32.6 (13.6)
Recurrent MDD, *n* (%)	137 (75.3)	120 (68.6)
Duration of current episode, mean (SD), months	15.1 (29.5)	20.2 (59.8)
Baseline MADRS score, mean (SD)	35.5 (4.0)	35.9 (4.1)

BMI, body mass index; ER, extended-release; MADRS, Montgomery-Åsberg Depression Rating Scale.

**Table 3.** Efficacy and health outcomes results (ITT population).

Characteristics	Placebo (*n* = 181)	Levomilnacipran ER 40–120 mg/day (*n* = 174)
Secondary efficacy outcome (MMRM)
SDS total score
Baseline, mean (SEM)	20.8 (0.4)	21.7 (0.4)
LS mean change (SE)	−8.2 (0.7)	−8.8 (0.7)
Additional efficacy outcomes (MMRM)
HAMD_17_ total score
Baseline, mean (SEM)	24.4 (0.3)	24.9 (0.3)
LS mean change (SE)	−9.2 (0.6)	−9.9 (0.7)
CGI-I total score
Value at Week 8 (SE)	2.7 (0.1)	2.5 (0.1)
CGI-S total score
Baseline, mean (SEM)	4.7 (0.0)	4.8 (0.0)
LS mean change (SE)	−1.3 (0.1)	−1.5 (0.1)
Health outcomes (LOCF)
SF-36 PCS
Baseline, mean (SEM)	51.1 (0.8)	51.6 (0.8)
LS mean change (SE)	−1.2 (0.7)	0.2 (0.7)
SF-36 MCS
Baseline, mean (SEM)	19.1 (0.7)	18.3 (0.7)
LS mean change (SE)	14.9 (1.2)	15.7 (1.2)

CGI-I, Clinical Global Impression-Improvement; CGI-S, Clinical Global Impressions-Severity; ER, extended-release; HAMD, Hamilton Rating Scale for Depression; ITT, intent-to-treat; MCS, Mental Component Summary; PCS, Physical Component Summary; SDS, Sheehan Disability Score; SE, standard error; SEM, standard error of the mean; SF-36, Short-Form 36 Health Survey.

### Efficacy outcomes

**Figure 1.**
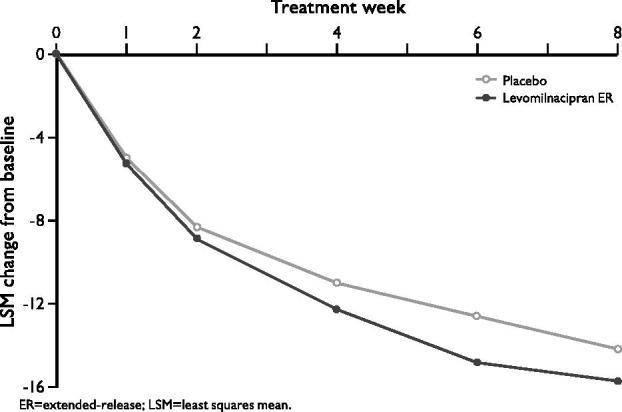
Change from baseline in MADRS total score (ITT population, MMRM).

On the primary efficacy endpoint, change from baseline to Week 8 in MADRS total score, numerically greater improvement was observed for patients in the levomilnacipran ER group (−15.7) compared with the placebo group (−14.2) (Figure 1); however, the between-group difference was not statistically significant (LSMD = −1.5, *p* = 0.249, MMRM). Sensitivity analyses using the LOCF approach (−14.8 vs −13.4, *p* = 0.260) and PMM model (data not shown) supported the primary results.

On the secondary efficacy measure, change from baseline to Week 8 in SDS total score, numerically greater change was noted in the levomilnacipran ER group (−8.8) compared with placebo (−8.2), but the difference did not reach statistical significance; similarly, numerically greater, but not statistically significant, differences were observed for levomilnacipran ER on additional efficacy measures (Table 3).

Response and remission rates at the end of double-blind treatment were numerically higher for the levomilnacipran ER group compared with placebo, but no statistically significant differences were observed between groups. MADRS response (≥50% reduction in total score from baseline) was achieved by 38.5% and 34.8% of levomilnacipran ER- and placebo-treated patients, respectively; CGI-I response (score ≤2) rates were 44.3% and 38.7% for patients treated with levomilnacipran ER and placebo, respectively. Remission (MADRS total score ≤10) was achieved in 25.3% of patients treated with levomilnacipran ER and 23.8% of placebo-treated patients.

No statistically significant differences were observed on the summary scales of the SF-36 health outcome measure (Table 3). Mean PCS baseline scores indicated no marked decrement in physical health in either treatment group at study entry. Mean baseline MCS scores indicated considerable mental health deficits in both levomilnacipran ER and placebo treatment groups; adjusted mean score increases were similar between groups.

### Safety outcomes

The mean duration of double-blind treatment for patients in the placebo and levomilnacipran ER groups was 51.0 and 49.7 days, respectively. The mean final daily levomilnacipran ER dose was 93.0 mg; the final daily dose was 40 mg/day for 17% of patients, 80 mg/day for 34% of patients, and 120 mg/day for 50% of patients.

#### Adverse events

No deaths were reported during this trial. Levomilnacipran ER was generally well tolerated. Overall, 62.6% of placebo-treated patients and 80.0% of levomilnacipran ER-treated patients reported at least one treatment-emergent adverse event (TEAE); the most common TEAEs (≥5% in either treatment group) are presented in Table 4. For placebo and levomilnacipran ER patients, 97% and 96% of TEAEs, respectively, were considered to be of mild or moderate intensity. TEAEs that were considered by the Investigator to be related to study drug occurred in 64% of levomilnacipran ER patients and 36% of placebo patients. The TEAEs that were reported in at least 5% of patients in the levomilnacipran ER group and at an incidence at least twice the rate of placebo were nausea, hyperhidrosis, dizziness, vomiting, and heart rate increased; ejaculation disorder and erectile dysfunction were also reported at this rate in male patients. TEAEs that led to the discontinuation of ≥2 patients in either treatment group were nausea, anxiety, and back pain.

**Table 4.** Most frequent (≥5% in any treatment group) double-blind treatment emergent adverse event (safety population).

Event	Placebo, % (*n* = 182)	Levomilnacipran ER 40–120 mg/day, % (*n* = 175)
Double-blind TEAEs
Nausea	3.3	17.1
Headache	12.1	16.0
Dry mouth	6.0	8.0
Hyperhidrosis	1.1	6.9
Insomnia	7.1	6.9
Dizziness	1.6	6.3
Vomiting	0.5	5.7
Heart rate increased	2.2	5.1
Upper respiratory tract infection	6.0	3.4
Ejaculation disorder^a^	0	7.9
Erectile dysfunction^a^	1.5	5.3

^a^Based on the number of males in the safety population (placebo = 66, levomilnacipran ER = 76). AE, adverse event; ER, extended-release; TEAE, treatment-emergent AE.

During double-blind treatment, no serious AEs (SAEs) were reported in the levomilnacipran ER group and two SAEs were reported by one patient (increased blood pressure and chest pain) in the placebo group. None of the TEAEs that led to premature discontinuation during double-blind treatment were SAEs and no SAEs were reported for any patient during the double-blind down-taper period.

#### Clinical laboratory values, vital signs, and physical changes

Changes in vital signs, clinical laboratory values, and ECGs were generally small and similar between treatment groups, and not considered clinically significant. For levomilnacipran ER patients vs placebo, slight mean changes were observed in alanine aminotransferase (ALT) (2.9 U/L vs −0.6 U/L), aspartate aminotransferase (AST) (0.2 U/L vs 2.2 U/L), and alkaline phosphatase (−1.5 U/L vs 6.2 U/L). No patient met Hy’s Law criteria (ALT or AST elevation ≥3 × UNL, total bilirubin elevation >2 × UNL, and alkaline phosphatase ≤2 × UNL)^28^.

Mean (SD) change in blood pressure and pulse rate (PR) were greater for patients in the levomilnacipran ER group compared with placebo (Table 5). TEAEs associated with changes in blood pressure and/or pulse rate occurred in one placebo patient and one levomilnacipran ER patient; none of these TEAEs was an SAE or led to discontinuation from the study and they resolved with continued use of the assigned treatment.

**Table 5.** Mean change in blood pressure and pulse rate (safety population).

Parameter, unit	Placebo (*n* = 182)		Levomilnacipran ER 40–120 mg/day (*n* = 175)	
	*n*	Mean (SD)	*n*	Mean (SD)
Supine systolic blood pressure, mmHg				
Baseline	181	119.5 (11.0)	174	118.7 (10.7)
Change at end of treatment	181	−0.6 (8.9)	174	2.8 (9.2)
Supine diastolic blood pressure, mmHg				
Baseline	181	76.1 (7.4)	174	75.8 (8.2)
Change at end of treatment	181	−0.3 (7.6)	174	3.3 (8.4)
Supine pulse rate, bpm				
Baseline	181	69.4 (7.7)	174	69.9 (9.0)
Change at end of treatment	181	−0.1 (8.2)	174	6.9 (9.7)

bpm, beats per minute; ER, extended-release.

A greater mean increase in ECG ventricular heart rate was observed in the levomilnacipran ER treatment group (12.7 bpm) relative to placebo (1.7 bpm). Greater increase in the QT interval corrected for heart rate using the Bazett formula (QTcB) was observed in the levomilnacipran ER group (10.7 ms) compared with placebo (0.4 ms); this increase was consistent with the observed increase in heart rate. Changes in the QT interval corrected for heart rate using the Fridericia formula (QTcF) were virtually identical for levomilnacipran ER (−1.5 ms) and placebo (−1.2 ms). A small mean decrease in PR interval was observed in the levomilnacipran ER group (−6.9 ms) compared to baseline, while placebo remained relatively unchanged (0.8 ms).

Mean changes from baseline in body weight were small and similar for levomilnacipran ER patients (−0.46 kg) and placebo patients (0.12 kg).

#### Arizona sexual experiences scale

The majority of male and female patients reported sexual dysfunction at baseline; the percentage of patients reporting sexual dysfunction at the double-blind treatment end-point decreased in all patient groups (Table 6). ASEX total scores showed similar small mean improvements for men and women in both the levomilnacipran ER and placebo treatment groups (Table 6).

**Table 6.** ASEX change in sexual dysfunction (safety population).

	Placebo			Levomilnacipran ER 40–120 mg/day		
	*n*	*n* (%)	Mean (SD)	*n*	*n* (%)	Mean (SD)
Percentage of patients with sexual dysfunction^a^						
Women	109			94		
Baseline sexual dysfunction		97 (89.0)			84 (89.4)	
Endpoint sexual dysfunction		71 (65.1)			68 (72.3)	
Men	65			71		
Sexual dysfunction at baseline		40 (61.5)			50 (70.4)	
Sexual dysfunction at endpoint		24 (36.9)			38 (53.5)	
Change in sexual dysfunction: ASEX total score						
Women
Baseline	104		21.8 (5.0)	87		23.0 (4.9)
Change at Week 8	94		−2.4 (5.3)	72		−2.3 (4.1)
Men
Baseline	61		18.6 (5.3)	67		18.8 (5.2)
Change at Week 8	54		−2.2 (4.3)	55		−1.1 (4.3)

^a^Categorical sexual dysfunction reports the number (%) of patients with sexual dysfunction at baseline and endpoint; sexual dysfunction = total ASEX ≥19, or an individual item score ≥5, or a score ≥4 on three individual items^44^; endpoint = last available double-blind post-baseline assessment. ASEX, Arizona Sexual Dysfunction Experience Scale; ER, extended-release.

#### Suicidal ideation and behavior

The incidence of suicidal ideation as measured by the C-SSRS was similar in the placebo and levomilnacipran ER treatment groups (22.1% and 23.6%, respectively). The majority of suicidal ideation reported in the placebo group (15%) and the levomilnacipran ER group (18%) were in the least severe category (‘wish to be dead’, with no active intent or plan). No patients in either treatment group completed suicide or had reports of suicidal behavior during double-blind treatment.

A TEAE of suicidal ideation was reported in one patient who received levomilnacipran ER for 23 days; the patient had a history of aborted suicide attempts. Increased severity of depression and suicidal ideation began on Day 19 and resulted in discontinuation from the study.

## Discussion

This Phase III trial evaluated the efficacy, safety, and tolerability of flexible doses of levomilnacipran ER (40–120 mg/day) compared with placebo in the treatment of MDD. Although levomilnacipran ER demonstrated numerically greater reduction from baseline in MADRS total score, the primary endpoint, the difference from placebo was not statistically significant. Similarly, numerically greater, but not statistically significant, change was seen for levomilnacipran ER compared with placebo on the secondary and additional efficacy measures.

The failure of levomilnacipran ER to demonstrate statistical superiority over placebo in this study is inconsistent with results from four positive, double-blind, placebo-controlled trials in the treatment of MDD^14–17^. Statistically significant differences for levomilnacipran ER relative to placebo were seen on the primary efficacy measure, MADRS total score change from baseline (LS mean [SE]) in: a 10-week, flexible-dose, Phase II trial of levomilnacipran ER 75–100 mg/day (placebo = −14.5 [0.56], levomilnacipran ER = −18.7 [0.56]; *p* < 0.0001) (EudraCT:2006-002404-34)^14^; an 8-week, flexible-dose, Phase III trial of levomilnacipran ER 40–120 mg/day (placebo = −12.2 [0.78], levomilnacipran ER = −15.3 [0.79]; *p* = 0.0051) (NCT01034462)^15^; an 8-week, fixed-dose, Phase III trial of levomilnacipran ER 40 mg/day and 80 mg/day (placebo = −11.3 [0.77], levomilnacipran ER 40 mg = −14.6 [0.79], *p* = 0.0027; levomilnacipran ER 80 mg = −14.4 [0.79], *p* = 0.0043) (NCT01377194)^17^; and an 8-week, fixed-dose Phase III trial of levomilnacipran ER 40 mg/day, 80 mg/day, and 120 mg/day (placebo = −11.6 [0.97], levomilnacipran ER 40 mg = −14.8 [0.99], *p* = 0.0186; levomilnacipran ER 80 mg = −15.6 [1.00], *p* = 0.0038; levomilnacipran ER 120 mg = −16.5 [1.02], *p* = 0.0005) (NCT00969709)^16^. Statistically significant differences were also observed on secondary efficacy measures, and many or all additional measures in all studies.

The most salient difference between the current trial and the positive studies just described was the magnitude of placebo response. The mean reduction from baseline in MADRS total score for levomilnacipran ER 40–120 mg in the current study was 15.7 points, which is comparable to the magnitude of change observed in the positive clinical trials. By comparison, in the current study patients receiving placebo experienced a mean MADRS total score reduction from baseline of 14.2 points, which is 2–3-points greater than the mean reductions observed for placebo groups in the positive levomilnacipran ER trials.

The increasing occurrence of robust placebo response in antidepressant clinical trials has been well documented^29–31^. This phenomenon is of particular concern because high placebo response, as seen in this trial, interferes with the sensitivity of the study to detect the efficacy of active treatment. In trials of marketed antidepressants submitted to the FDA, fewer than half demonstrated statistically superior efficacy for the active compound^32,33^. Meta-analyses suggest that high placebo response, rather than poor response to medication, explains much of the variability in drug–placebo differences in clinical studies^34,35^.

High and variable placebo response rates contribute to the likelihood of ambiguous findings in antidepressant clinical trials, which may hinder the development of new antidepressant treatment options. Several factors have been evaluated to determine if specific aspects of study design and conduct influence placebo response and treatment effect in clinical trials of antidepressants. Baseline depression severity, flexible- vs fixed-dose regimens, where and when the study was conducted, study duration, patient age, and the permitted use of sedatives or anxiolytics are among the factors that have been suggested as contributors to high placebo response^29,35,36^.

A greater likelihood of receiving placebo, greater severity of baseline depression, and earlier year of study publication appear to be associated with greater antidepressant–placebo separation at the end of treatment^35,36^. Analyses of fixed- vs flexible-dosage schedules on treatment effects have yielded equivocal results. Khan *et al*.^37,38^ reported success rates of 59.6% for MDD trials with a flexible-dose design vs 31.4% for fixed-dose trials; conversely, an analysis by Khin *et al*.^35^ found a slightly higher success rate in fixed-dose (57%) vs flexible-dose (51%) trials. Generally, fixed- vs flexible-dosage schedules, trial duration, and patient age do not routinely appear to influence trial outcome^36^. The use of a placebo run-in phase, restricting study design to two treatment arms with 1:1 randomization, and limiting patient expectation of improvement through explicit consent practices have also been posited as potential ameliorators of high placebo response, but investigations of these factors have yielded conflicting outcomes^29,35,36^ and more study is warranted.

Based on these findings, the study design used in the current trial should have minimized a majority of the factors that have been mentioned as possible reasons for high placebo response. Single-blind placebo was administered during the 1-week, placebo run-in period to minimize placebo response and allow a preliminary evaluation of patient compliance with investigational product dosing. After the screening period, patients were randomized 1:1 to only levomilnacipran ER or placebo. Additionally, the population consisted of patients whose mean baseline MADRS total score (36) exceeded the threshold typically used to delineate severe depression (baseline MADRS total ≥30)^39^, which would suggest a study population with highly symptomatic and at least moderately severe depression. Since more severe depression is one of the factors that has been shown to be significantly related to positive antidepressant trial outcome^35,36,40,41^, baseline level of depression severity may not be an important factor in the outcome of the current trial.

It has been suggested that limiting the number of rating scales and shortening the length of study visits may help minimize the high placebo effect^42^. Although the primary objective of this study was to evaluate the efficacy, safety, and tolerability of levomilnacipran ER vs placebo, several additional efficacy measures were used in this study to evaluate MDD-associated impairment that occurs across several life and health domains. While it is not possible to determine if this influenced the rate of placebo response, the inclusion of additional efficacy measures to evaluate functional as well as symptomatic improvement was warranted. Of note, in positive levomilnacipran ER clinical studies, the use of multiple efficacy measures did not appear to influence the ability to detect a positive result on the primary measure^14–17^. Additionally, the rate of response (MADRS ≥50% improvement from baseline) for levomilnacipran ER patients was slightly lower relative to other levomilnacipran ER studies (39% in this study vs 42–47% in other studies), which suggests that there may have been additional undetected factors that influenced study results. While the negative finding on the primary outcome in this study appears to be related to a high rate of placebo response, the reason for this and other contributing factors remains unclear.

Similar to findings from the positive levomilnacipran ER studies mentioned, levomilnacipran ER was generally well tolerated in the current study. The most common TEAEs were typical of agents that selectively inhibit serotonin and norepinephrine reuptake^43^, and most were judged to be mild-to-moderate in intensity. No SAEs, suicides, or suicide attempts occurred during the study. Levomilnacipran ER was weight neutral, an important finding in light of the high percentage of patients who rate weight gain as a bothersome AE associated with antidepressant use^8^.

Sexual dysfunction is another common and troublesome AE associated with depression and antidepressant use. This is the only levomilnacipran ER trial reported to date that has used the ASEX, a standardized measure of sexual function, to prospectively and categorically assess sexual experiences. Patients receiving levomilnacipran ER or placebo showed small improvement in ASEX total score after 8 weeks of treatment. No decrease in sexual function and change similar to placebo are relevant findings pertaining to the good tolerability profile of levomilnacipran ER. Using an alternate sexual functioning measure, sexual dysfunction TEAEs (male patients only) were reported for levomilnacipran ER patients vs placebo patients: ejaculation disorder (7.9% vs 0%) and erectile dysfunction (5.3% vs 1.5%).

Although a high rate of placebo response contributed to the lack of statistical separation between drug and placebo observed in this flexible-dose trial of levomilnacipran ER, no specific methodological or design issue has been identified as an explicit reason for the negative outcome. Limitations of the study include the short duration of double-blind treatment, inclusion and exclusion criteria that may limit generalizability, and no active comparator. In light of the highly successful clinical development program for levomilnacipran ER, this negative study appears to be an anomaly, albeit one that has occurred more frequently in recent antidepressant clinical trials.

## Conclusion

In this study, numerically greater improvements were consistently observed across efficacy measures, suggesting a trend toward improvement for levomilnacipran ER relative to placebo, but between-treatment differences were not statistically significant. Levomilnacipran ER was generally safe and well tolerated. A relatively low incidence of sexual AEs was reported, in addition to small improvements in sexual functioning, using a prospectively defined sexual dysfunction outcome measure; this result is relevant to the overall tolerability profile of levomilnacipran ER, since sexual dysfunction is commonly associated with depression and antidepressant use. The findings from this negative trial should be viewed within the context of the four robustly positive levomilnacipran ER placebo-controlled trials and the current clinical trial environment in which nearly half of all placebo-controlled antidepressant trials do not demonstrate significant antidepressant separation from placebo on the primary efficacy outcome measure.

## Transparency

### Declaration of financial/other relationships

CG, WMG, and CC are employees of Forest Research Institute, a subsidiary of Forest Laboratories, Inc.

## Previous presentation

Gommoll C, Bose A, Li H, Edwards J. A randomized double-blind, placebo-controlled, flexible-dose study of levomilnacipran in patients with major depressive disorder. Poster presented at: the 24th Annual US Psychiatric and Mental Health Congress; Las Vegas, Nevada, November 7–10, 2011.
